# Supplementary material for: Current situation of the hospitalization of persons without family in Japan and related medical challenges
Source: PLoS One. 2023 Jun 2;18(6):e0276090. doi: 10.1371/journal.pone.0276090 (PMC10237481; doi:10.1371/journal.pone.0276090)
Supplement: S1 Questionnaire — (DOCX) [file pone.0276090.s002.docx]

**S1. Questionnaire**

*Question 1 is about the medical institution where you work. Please answer the following questions.

1. Please tell us about your medical institution.

1-1. Please indicate the location (prefecture and city) of your medical institution.

1. prefecture ( )

2. city ( )

1-2. Please indicate the category of your medical institution.

1. General hospital

2. Hospital with long-term care beds

3. Advanced treatment hospital

4. Regional medical support hospital

1-3. Please indicate the number of beds in your medical institution.

1. 20–49

2. 50–99

3. 100–199

4. 200–399

5. 400 or more beds

*Question 2 is about the hospitalization and medical care status for persons without family. Please answer the following questions.

2. We would like to ask you about your institution’s hospitalization and medical treatment status for persons without family.

2-1. Please enumerate the number of hospitalizations in a year for persons without family.

In one year, there have been ( ) cases.

2-2. Please indicate the challenges of dealing with hospitalization and medical care for persons without family (multiple responses allowed).

1. Lack of emergency contact information

2. Matters related to hospitalization plan

3. Matters related to the preparation of supplies needed during hospitalization

4. Matters related to hospitalization expenses

5. Matters related to discharge support

6. (In the event of death) Matters related to the retrieval of the body and belongings and funeral services

7. Decision-making related to medical care

8. Others ( )

2-3. What is the decision-making process for medical care for persons without family, if the person's will cannot be confirmed during the decision-making for medical care? (Multiple responses allowed)

1. Decisions made according to existing manuals and guidelines

2. Decisions made by the medical and care team

3. Decisions made at conferences

4. Decisions made by the ethics committee

5. Decisions made by the attending physician

6. Decisions made by the patient's acquaintances and friends

8. Others ( )

3. We would like to inquire about your medical institution's response based on the “Guidelines for the Hospitalization of Persons without Family and Support for Persons with Difficulty in Decision-Making Regarding Medical Care” (hereinafter referred to as “Guidelines”).

Has your hospital taken any action based on the “Guidelines”?

1. We have taken action based on the Guidelines.

2. We have never responded according to the Guidelines.

3. We do not know about the Guidelines.
