# Supplementary material for: Current situation of the hospitalization of persons without family in Japan and related medical challenges
Source: PLoS One. 2023 Jun 2;18(6):e0276090. doi: 10.1371/journal.pone.0276090 (PMC10237481; doi:10.1371/journal.pone.0276090)
Supplement: S1 Table — (DOCX) [file pone.0276090.s003.docx]

**S2 Table. Region and hospital type**

|  | General hospitals | Hospitals with long-term care beds | Advanced treatment hospitals | Regional medical care support hospitals |
| --- | --- | --- | --- | --- |
| Local area | 322 (41.0) ^※^ | 401 (51.0) | 15 (1.9) | 48 (6.1) |
| Tokyo area | 115 (48.7) | 100 (42.4) | 5 (2.1) | 16 (6.8) |
| Osaka area | 65 (41.1) | 75 (47.5) | 4 (2.5) | 14 (8.9) |
| Nagoya area | 16 (30.8) | 30 (57.7) | 0 (0) | 6 (11.5) |
| ※ frequency (percentage) | |  |  |  |
