# Supplementary material for: Current situation of the hospitalization of persons without family in Japan and related medical challenges
Source: PLoS One. 2023 Jun 2;18(6):e0276090. doi: 10.1371/journal.pone.0276090 (PMC10237481; doi:10.1371/journal.pone.0276090)
Supplement: S2 Table — (DOCX) [file pone.0276090.s004.docx]

**S3 Table. Hospital type and number of beds**

|  | 20-49 | 50-99 | 100-199 | 200-399 | 400- |
| --- | --- | --- | --- | --- | --- |
| General hospitals | 76 (14.6) | 138 (26.6) | 161 (31.0) | 100 (19.3) | 44 (8.5) |
| Hospitals with long-term care beds | 34 (5.6) | 158 (25.9) | 294 (48.1) | 107 (17.5) | 18 (2.9) |
| Advanced treatment hospitals | 0 (0) | 0 (0) | 0 (0) | 0 (0) | 24 (100) |
| Regional medical care support hospitals | 0 (0) | 0 (0) | 0 (0) | 37 (44.1) | 47 (55.9) |
