# Supplementary material for: Current situation of the hospitalization of persons without family in Japan and related medical challenges
Source: PLoS One. 2023 Jun 2;18(6):e0276090. doi: 10.1371/journal.pone.0276090 (PMC10237481; doi:10.1371/journal.pone.0276090)
Supplement: S3 Table — (DOCX) [file pone.0276090.s005.docx]

**S4 Table. Number of beds and region**

|  | Local area | Tokyo area | Osaka area | Nagoya area |
| --- | --- | --- | --- | --- |
| 20–49 | 79 (69.3) | 17 (14.9) | 14 (12.3) | 4 (3.5) |
| 50–99 | 194 (65.5) | 57 (19.3) | 36 (12.2) | 9 (3.0) |
| 100–199 | 285 (62.1) | 92 (20.0) | 65 (14.2) | 17 (3.7) |
| 200–399 | 157 (63.3) | 51 (20.5) | 26 (10.5) | 14 (5.7) |
| 400+ | 78 (58.6) | 27 (20.3) | 19 (14.3) | 9 (6.8) |
