# Supplementary material for: Current situation of the hospitalization of persons without family in Japan and related medical challenges
Source: PLoS One. 2023 Jun 2;18(6):e0276090. doi: 10.1371/journal.pone.0276090 (PMC10237481; doi:10.1371/journal.pone.0276090)
Supplement: S4 Table — (DOCX) [file pone.0276090.s006.docx]

**S5 Table. Comparison between nurses’ and medical social workers’ responses**

|  |  | Nurses ^a^ (n=301) | |  | Medical social workers ^a^ (n=820) | |  |
| --- | --- | --- | --- | --- | --- | --- | --- |
|  |  | ｎ | ％ ^a^ |  | n | ％ ^a^ | *P** |
| **Region** |  |  |  |  |  |  |  |
|  | Local Area | 200 | 66.4 |  | 503 | 61.3 | 0.597 |
|  | Tokyo area | 50 | 16.6 |  | 167 | 20.4 |  |
|  | Osaka area | 37 | 12.3 |  | 110 | 13.4 |  |
|  | Nagoya area | 11 | 3.7 |  | 32 | 3.9 |  |
|  | Missing value | 3 | 1.0 |  | 8 | 1.0 |  |
| **Hospital type** | |  |  |  |  |  |  |
|  | General hospitals^b^ | 136 | 45.2 |  | 326 | 39.8 | 0.005 |
|  | Hospitals with long-term care beds | 146 | 48.5 |  | 390 | 47.6 |  |
|  | Advanced treatment hospitals | 1 | 0.3 |  | 21 | 2.6 |  |
|  | Regional medical care support hospitals | 10 | 3.3 |  | 66 | 8.0 |  |
|  | Missing value | 8 | 2.7 |  | 17 | 2.1 |  |
| **Establishing entity** | |  |  |  |  |  |  |
|  | National | 228 | 75.7 |  | 568 | 69.3 | 0.078 |
|  | Public organization | 12 | 4.0 |  | 46 | 5.6 |  |
|  | Private corporation or individual | 59 | 19.6 |  | 169 | 20.6 |  |
|  | Missing value | 2 | 0.7 |  | 18 | 2.2 |  |
| **Number of beds** | |  |  |  |  |  |  |
|  | 20–49 | 70 | 23.3 |  | 31 | 3.8 | <0.001 |
|  | 50–99 | 89 | 29.6 |  | 168 | 20.5 |  |
|  | 100–199 | 87 | 28.9 |  | 325 | 39.6 |  |
|  | 200–399 | 39 | 13.0 |  | 184 | 22.4 |  |
|  | 400+ | 15 | 5.0 |  | 106 | 12.9 |  |
|  | Missing value | 1 | 0.3 |  | 6 | 0.7 |  |
| **Situations that were difficult to deal with during the hospitalization of a person without family (multiple responses)** | | | |  |  |  |  |
|  | Emergency contact information | 197 | 65.4 |  | 629 | 76.7 | - |
|  | Matters related to hospitalization plans | 81 | 26.9 |  | 216 | 26.3 |  |
|  | Matters related to supplies needed during hospitalization | 140 | 46.5 |  | 485 | 59.1 |  |
|  | Matters related to hospitalization expenses | 126 | 41.9 |  | 547 | 66.7 |  |
|  | Matters related to discharge support | 137 | 45.5 |  | 566 | 69.0 |  |
|  | Matters related to the retrieval of the body and belongings and funeral services | 142 | 47.2 |  | 482 | 58.8 |  |
|  | Decision-making related to medical care | 179 | 59.5 |  | 567 | 69.1 |  |
| **Decision-making process for medical care for persons without family (multiple responses)** | | | |  |  |  |  |
|  | Decisions made according to manuals and guidelines | 80 | 26.6 |  | 257 | 31.3 | - |
|  | Decisions made by the medical care team | 102 | 33.9 |  | 411 | 50.1 |  |
|  | Decisions made at conferences | 91 | 30.2 |  | 383 | 46.7 |  |
|  | Decisions made by the ethics committee | 36 | 12.0 |  | 165 | 20.1 |  |
|  | Decisions made by the attending physician | 126 | 41.9 |  | 340 | 41.5 |  |
|  | Decisions made by patient's acquaintances and friends | 8 | 2.7 |  | 12 | 1.5 |  |
|  | Decisions made by the nurse in charge | 12 | 4.0 |  | 47 | 5.7 |  |
|  | Decisions made by the medical social worker | 63 | 20.9 |  | 154 | 18.8 |  |
| **Use of the Guidelines^c^** | |  |  |  |  |  |  |
|  | We have never responded according to the Guidelines | 35 | 11.6 |  | 200 | 24.4 | <0.001 |
|  | We have taken action in accordance with the Guidelines | 130 | 43.2 |  | 399 | 48.7 |  |
|  | We do not know about the Guidelines | 124 | 41.2 |  | 207 | 25.2 |  |
|  | Missing value | 12 | 4.0 |  | 14 | 1.7 |  |
| ^a^ Excluded respondents with both roles (n=81) | | | |  |  |  |  |
| ^b^ Percentage divided by total number | |  |  |  |  |  |  |
| ^c^ Hospitals without beds for long-term care | | |  |  |  |  |  |
| ^d^ Guidelines for hospitalization of persons without family and support for persons with decision-making difficulties | | | | | | | |
| *p < .05 | | | | | | | |
