# Supplementary material for: Current situation of the hospitalization of persons without family in Japan and related medical challenges
Source: PLoS One. 2023 Jun 2;18(6):e0276090. doi: 10.1371/journal.pone.0276090 (PMC10237481; doi:10.1371/journal.pone.0276090)
Supplement: S5 Table — (DOCX) [file pone.0276090.s007.docx]

**S6 Table. Comparison between the number of hospitalizations of persons without family reported by nurses and medical social workers (approximate number per year)**

|  |  |  |  | |  | |  | |  | Percentiles | | | *P** |
| --- | --- | --- | --- | --- | --- | --- | --- | --- | --- | --- | --- | --- | --- |
|  |  | n | Min | | Max | | Mean (SD) | | Median | 25 | 50 | 75 |  |
| Nurses |  | 202 | 1 | | 120 | | 7(13) | | 3 | 2 | 3 | 10 | <0.001 |
| Medical social workers |  | 650 | 0.5 | | 2000 | | 20(94) | | 6 | 3 | 6 | 15 |  |
| *p < .05 |  |  | |  | |  | |  |  |  |  |  |  |
