# Supplementary material for: Current situation of the hospitalization of persons without family in Japan and related medical challenges
Source: PLoS One. 2023 Jun 2;18(6):e0276090. doi: 10.1371/journal.pone.0276090 (PMC10237481; doi:10.1371/journal.pone.0276090)
Supplement: S6 Table — (DOCX) [file pone.0276090.s008.docx]

**S7 Table. Number of hospitalizations of persons without family excluding outliers >200 ^a^ (approximate number per year)**

|  |  |  |  |  |  |  | Percentiles | | | *P** |
| --- | --- | --- | --- | --- | --- | --- | --- | --- | --- | --- |
|  |  | n | Min | Max | Mean (SD) | Median | 25 | 50 | 75 |  |
| **Total** |  | 946 | 0.5 | 150 | 11 (18) | 5 | 2 | 5 | 12 |  |
| **Region ^b^** |  |  |  |  |  |  |  |  |  |  |
| Local area |  | 604 | 0.5 | 120 | 8(12) | 5 | 2 | 5 | 12 | <0.001 |
| Tokyo area |  | 169 | 1 | 150 | 20(28) | 10 | 5 | 10 | 20 |  |
| Osaka area |  | 130 | 1 | 150 | 14(21) | 6.5 | 3 | 7 | 15 |  |
| Nagoya area |  | 36 | 1 | 75 | 10(14) | 5 | 2 | 5 | 12.5 |  |
| Missing value |  |  | - | - | - | - | - | - | - |  |
| **Hospital type ^b^** |  |  |  |  |  |  |  |  |  |  |
| General hospitals |  | 392 | 1 | 140 | 12(18) | 5 | 2 | 5 | 15 | <0.001 |
| Hospitals with long-term care beds |  | 459 | 0.5 | 150 | 8(15) | 4 | 2 | 4 | 10 |  |
| Advanced treatment hospitals |  | 14 | 10 | 118 | 26(28) | 19 | 12 | 19 | 25 |  |
| Regional medical care support hospitals |  | 61 | 1 | 150 | 24(27) | 15 | 10 | 15 | 30 |  |
| Missing value |  |  | - | - | - | - | - | - | - |  |
| **Number of beds ^c^** |  |  |  |  |  |  |  |  |  |  |
| 20–49 |  | 79 | 1 | 38 | 5(7) | 2 | 1 | 2 | 6 | <0.001 |
| 50–99 |  | 216 | 1 | 150 | 7(13) | 5 | 3 | 8 | 15 |  |
| 100–199 |  | 367 | 0.5 | 120 | 15(10) | 5 | 2.5 | 5 | 10 |  |
| 200–399 |  | 188 | 1 | 150 | 14(2) | 8 | 4 | 8 | 16 |  |
| 400+ |  | 90 | 1 | 120 | 24(25) | 15 | 10 | 15 | 30 |  |
| Missing value |  |  | - | - | - | - | - | - | - |  |
| ^a^ Outliers were calculated by Smirnov-Grubbs test. | | | | | | | | | | |
| ^b^ Number of individual hospitalizations, excluding missing values, compared between groups by Kruskal–Wallis test. | | | | | | | | | | |
| ^c^ Number of individual hospitalizations, excluding missing values, compared between groups by Jonckheere–Terpstra test. | | | | | | | | | | |
| *p < .05 |  |  |  |  |  |  |  |  |  |  |
