# Supplementary material for: Current situation of the hospitalization of persons without family in Japan and related medical challenges
Source: PLoS One. 2023 Jun 2;18(6):e0276090. doi: 10.1371/journal.pone.0276090 (PMC10237481; doi:10.1371/journal.pone.0276090)
Supplement: S7 Table — (DOCX) [file pone.0276090.s009.docx]

**S8 Table. Comparison of response rates by region**

|  |  | Distribution | Collection | Response rate (%) |
| --- | --- | --- | --- | --- |
| **Region** |  |  |  |  |
|  | Local Area | 2402 | 797 | 33.2 |
|  | Tokyo area | 851 | 245 | 28.8 |
|  | Osaka area | 551 | 162 | 29.4 |
|  | Nagoya area | 196 | 53 | 27.0 |
