# Supplementary material for: Current situation of the hospitalization of persons without family in Japan and related medical challenges
Source: PLoS One. 2023 Jun 2;18(6):e0276090. doi: 10.1371/journal.pone.0276090 (PMC10237481; doi:10.1371/journal.pone.0276090)
Supplement: S8 Table — (DOCX) [file pone.0276090.s010.docx]

**S9 Table. Comparison of missing values by region**

|  |  | Hospital type | |  | Establishing  entity | |  | Number of beds | |  | Use of  the Guidelinesc | |
| --- | --- | --- | --- | --- | --- | --- | --- | --- | --- | --- | --- | --- |
|  |  | n | % |  | n | % |  | n | % |  | n | % |
| **Region** | |  |  |  |  |  |  |  |  |  |  |  |
|  | Local Area (n=797) | 11 | 1.4 |  | 4 | 0.5 |  | 4 | 0.5 |  | 21 | 2.6 |
|  | Tokyo area (n=245) | 9 | 3.7 |  | 1 | 0.4 |  | 1 | 0.4 |  | 7 | 2.9 |
|  | Osaka area (n=162) | 4 | 2.4 |  | 1 | 0.6 |  | 2 | 1.2 |  | 3 | 1.9 |
|  | Nagoya area (n=53) | 1 | 1.9 |  | 0 | 0 |  | 0 | 0 |  | 0 | 0 |
