# Supplementary material for: Current situation of the hospitalization of persons without family in Japan and related medical challenges
Source: PLoS One. 2023 Jun 2;18(6):e0276090. doi: 10.1371/journal.pone.0276090 (PMC10237481; doi:10.1371/journal.pone.0276090)
Supplement: S9 Table — (DOCX) [file pone.0276090.s011.docx]

**S10 Table. Comparison of missing values by hospital type**

|  |  | Hospital type | | Establishing  entity | | Number of beds | | Use of  the Guidelinesc | |
| --- | --- | --- | --- | --- | --- | --- | --- | --- | --- |
|  |  | n | % | n | % | n | % | n | % |
| **Hospital type** | |  |  |  |  |  |  |  |  |
|  | General hospitalsb (n=522) | 4 | 0.8 | 2 | 0.4 | 3 | 0.6 | 12 | 2.3 |
|  | Hospitals with long-term care beds (n=612) | 6 | 1.0 | 0 | 0 | 1 | 0.2 | 16 | 2.6 |
|  | Advanced treatment hospitals (n=24) | 0 | 0 | 0 | 0 | 0 | 0 | 0 | 0 |
|  | Regional medical care support hospitals (n=84) | 0 | 0 | 0 | 0 | 0 | 0 | 1 | 1.2 |
